# [Expression of Concern] Lysine demethylase 2A promotes the progression of ovarian cancer by regulating the PI3K pathway and reversing epithelial-mesenchymal transition

**DOI:** 10.3892/or.2025.8971

**Published:** 2025-08-08

**Authors:** Dan-Hua Lu, Jiang Yang, Li-Kun Gao, Jie Min, Jian-Ming Tang, Ming Hu, Yang Li, Su-Ting Li, Jing Chen, Li Hong

**Affiliations:** Department of Gynaecology and Obstetrics, Renmin Hospital of Wuhan University, Wuhan, Hubei 430060, P.R. China; Department of Pathology, Molecular Diagnostics Laboratory, University of Michigan, Ann Arbor, MI 48109, USA

Oncol Rep 41: 917–927, 2019; DOI: 10.3892/or.2018.6888

Following the publication of this paper, it was drawn to the Editor's attention by a concerned reader that one of the colony-formation assay images shown in Fig. 2D for the SKOV3 experiments was strikingly similar to the ‘SKOV3/0 µM LY294002’ image shown in Fig. 5E, if one of the dishes were to be rotated 90°. Upon performing an independent analysis of the data in this paper in the Editorial Office, it also came to light that the data shown for the MMP9 blot (A2780 cell line) in Fig. 4E was strikingly similar to the p-mTOR blot (again for the A2780 cell line) in Fig. 5B, suggesting that one of these figures had been assembled incorrectly.

The authors were contacted by the Editorial Office to offer an explanation for these apparent anomalies in the presentation of the data in this paper; however, up to this time, no response from them has been forthcoming. Owing to the fact that the Editorial Office has been made aware of potential issues surrounding the scientific integrity of this paper, we are issuing an Expression of Concern to notify readers of this potential problem while the Editorial Office continues to investigate this matter further.

